# A Metachronous splenic metastases from esophageal cancer: a case report

**DOI:** 10.1186/1477-7819-9-105

**Published:** 2011-09-16

**Authors:** Ivan Botrugno, Vassili Jemos, Lorenzo Cobianchi, Giacomo Fiandrino, Silvia Brugnatelli, Vittorio Perfetti, Alessandro Vercelli, Marcello Maestri, Paolo Dionigi

**Affiliations:** 1 Department of Surgery, Fondazione IRCCS Policlinico San Matteo and University of Pavia, Pavia, Italy; 2 Department of Pathology Anatomy, Fondazione IRCCS Policlinico San Matteo and University of Pavia, Pavia, Italy; 3 Department of Internal Medicine, Fondazione IRCCS Policlinico San Matteo and University of Pavia, Pavia, Italy; 4 Department of Radiology, Fondazione IRCCS Policlinico San Matteo and University of Pavia, Pavia, Italy

**Keywords:** splenic metastases, esophageal cancer, splenic abscesses

## Abstract

The spleen is an infrequent site for metastatic lesions, and solitary splenic metastases from squamous cell carcinoma of the esophagus are very rare: only 4 cases have been reported thus far. These lesions are whitish nodules that are macroscopically and radiologically similar to primary splenic lymphomas. We report a case of metachronous splenic metastases from esophageal cancer and multiple splenic abscesses, which developed nine months after apparently curative esophagectomy without adjuvant chemotherapy. The patient underwent splenectomy dissection followed by adjuvant chemotherapy, but liver and skin metastases developed, and the patient died 9 months later.

## Background

Splenic metastases from solid malignancies generally occur within a setting of extensive multiorgan involvement. Over half of all patients with metastatic disease involving five or more organs have lesions in the spleen (often microscopic), and these patients represent 2-7% of those who die from end-stage metastatic disease [1,2]. It is much less common to find the spleen as the sole site of metastatic spread. In cases that have been reported, the splenic metastases were synchronous or metachronous lesions with patterns ranging from micro- or macronodular to diffuse infiltration of both the white and red pulp [3,4]. It is unclear why early splenic metastases are so uncommon, but several of the organ's features are suspected to contribute to their rarity. They include mechanical factors, such as the continuous nature of splenic blood flow, the rhythmic contraction of the splenic capsule, the acute angle at which the splenic artery branches from the celiac artery, and the absence in the spleen of afferent lymphatic vessels. It has also been suggested that the splenic microenvironment exerts an inhibitory effect on metastases and that the organ may produce a factor that inhibits the proliferation of non-hematopoietic cells [4-6]. Radiologically, metastatic lesions of the spleen resemble primary tumors involving this organ (e.g., lymphomas). Their differential diagnosis is based essentially on fine-needle aspiration cytology [FNAC] or surgical pathology. Immunostaining can reveal the origin of the lesion: epithelial (cytokeratins), melanocytic (S-100, HMB-45), or lymphatic (B/T-cell line CD antigens) [7].

Thus far, 93 cases of solitary splenic metastases have been reported in the literature. Most are secondary to colorectal (20 cases), ovarian (18), or lung (10) cancers, and adenocarcinoma primaries are more common than epidermoid tumors [4,8]. Only four cases of solitary metachronous metastases to the spleen in patients with squamous cell carcinoma (SCC) of the oesophagus have been documented so far [9-14] (Table 1). This report describes a case of metachronous metastases from esophageal SCC that were associated with multiple splenic abscesses.

**Table 1:** Details of all cases of splenic metastases from esophageal cancer reported in literature

Author	**Age of pt**.	TNM	Treatment	Time to onset of spleen mts	Symptoms	Treatment of metastases	Findings	Follow-up
**Sanyal et al**	25	pT3N1Mx	Transhiatal esophagectomy	15 months	Continuous, dull aching, epigastric and left hypocondrium pain radiating to back, anorexia and weight loss	Splenectomy, distal pancreasectomy, splenic flexure colon resection	Keratinizing squamous cell carcinoma infiltrating the pancreas	After 7 months doing well

**Vyas et al**	63	pT3N1Mx	Transhiatal esophagectomy	11 months	Persistent pain and vague fullness in the left hypocondrium	Systemic chemiotherapy with cisplatin and 5FU	FNAB: metastatic squamous cell carcinoma	Death after eleven months

**Hester**	65	T3N2M1	Sistemic chemiotherapy Oxaliplatin and capecitabine	9 months	Left flank pain radiating to the groin and ipovolemic shock for spleen spontaneous rupture	Splenic artery embolization		

**Cavanna**	50	T3N1M1	Systemic chemiotherapy with cisplatin and 5FU in continuous infusione (96 H)	synchronous	Vague discomfort in upper abdomen quadrant	Sistemic chemiotherapy	FNAB: metastatic squamous cell carcinoma	Reduction of the spleen lesion about 50%

**Kimura et al**	58	T3N0M0	Transhiatal esophagectomy	6 months	Vague discomfort in upper abdomen quadrant	Splenic artery embolization followed by splenectomy	Keratinizing squamous cell carcinoma	

### Case Presentation

A 59-year-old man was referred to the general surgery department of our hospital for a one-month history of progressive dysphagia for solids, which was not associated with malnutrition or significant weight loss. The patient had recently undergone esophagogastroduodenoscopy in another hospital, which revealed a bleeding, ulcerative lesion in the middle third of the esophagus, but no biopsy had been collected. The medical past history included COPD diagnosed in 1999 and a myocardial infarction in 2002. The patient had smoked approximately 25 cigarettes per day for several years.

Physical examination was unremarkable. Computed tomography (CT) of the chest and abdomen revealed stenosis involving a 5-cm segment of the middle third of the esophagus with no other lesions in the thoracic or abdominal organs. Barium studies disclosed a swelling in the esophageal wall 7 cm above the cardia with an ulcerative pattern, which reduced the diameter of the lumen to 5 mm. An endoscopic biopsy of the oesophageal mass demonstrated poorly differentiated (G3) squamous cell carcinoma.

Mid-distal esophagectomy was performed with oesophagogastric anastomosis and gastric tube reconstruction. Pathological examination of the surgical specimen confirmed the biopsy diagnosis of poorly differentiated (G3) SCC. The tumor, which measured 3 cm of length, had infiltrated the oesophageal wall and the surrounding paraesophageal fat. Surgical margins were tumor-free, as the seven perigastric limph-nodes dissected (pT3 N0). The postoperative period was quite unremarkable, and a contrast enhanced x-ray obtained on the 9th POD showed normal esophageal and gastric transit. On the 14th POD, the patient was discharged with an oncology referral for routine medical follow-up.

**Figure 1:**
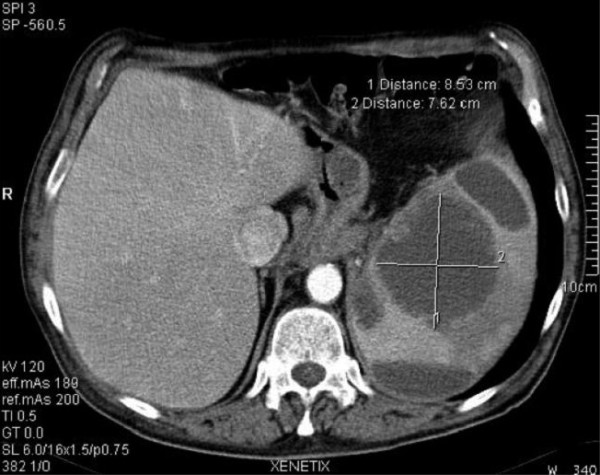
**CT-scan showing the largest site of splenic involvement: 8,5 × 7,6 cm**.

**Figure 2:**
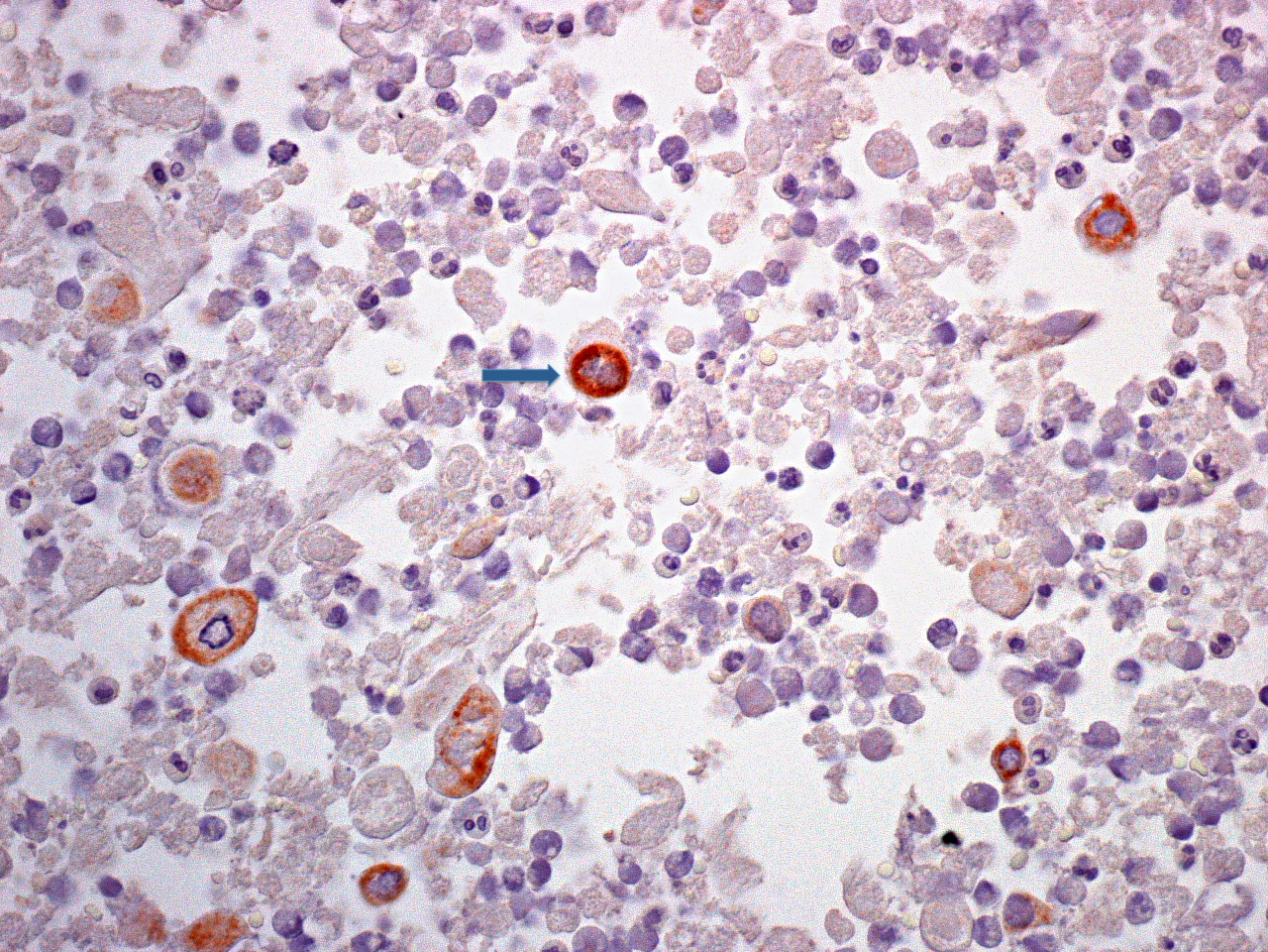
**Immunohistochemistry for pan-cytokeratin (KL-1 tag with arrow) on fine-needle aspirate from the splenic lesion highlights scattered positive cells (SABC method, 200×)**.

Nine months after the operation, CT and esophagogastroduodenoscopy were repeated. The imaging study revealed mild splenomegaly with multiple nonspecific nodules within the organ (Figure 1). The patient was virtually asymptomatic with the exception of a vague sensation of mild discomfort in the left upper quadrant of the abdomen. FNAC of the spleen revealed a pattern of numerous inflammatory cells admixed with large cells displaying immunohistochemical positivity for several cytokeratins (Figure 2). The specimen was Gram stain-negative. A bone-marrow biopsy was negative for metastatic involvement. The diagnosis was isolated metastases of the spleen with inflammatory and necrotic alterations.

The patient was referred to our centre for splenectomy, which was performed as a routine procedure to role out, also, a spontaneous rupture of the spleen. On 12^th ^December 2007, the patient had transabdominal total splenectomy with splenic and celiac artery lymph node dissection.

The postoperative course was uneventful. On the 7^th ^postoperative day, Doppler ultrasonography revealed portal-tree patency with no signs of thrombosis. Ten days later, the patient was discharged with a stable platelet count (780,000/mm^3^), Hb 10.9 g/dL, and a WBC count of 16,500/mm^3^.

**Figure 3:**
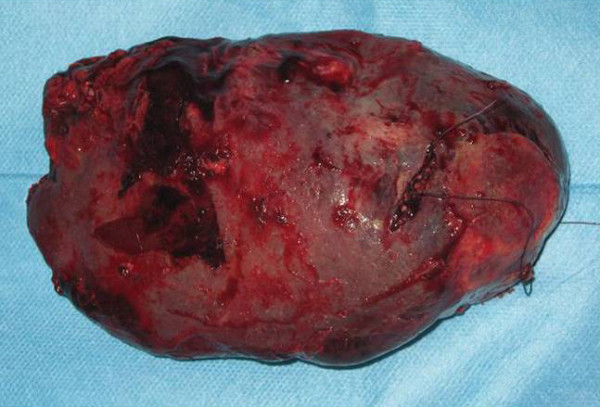
**Spleen; surgical specimen: 17 × 11 × 6 cm, weight: 600 g**.

Pathological examination of the spleen described multiple nodules containing medium to large-sized cells, some of which were keratinized. The nodules were mostly solid with areas of central necrosis (Figure 3). The findings were consistent with metastases of SCC. Thereafter, the patient was referred to the oncology department of our hospital, where he received two 3-day cycles (separated by a 3-week interval) of systemic chemotherapy based on 5-fluorouracil (800 mg/day IV) and cisplatin (20 mg/day). Three months after the splenectomy, multiple liver metastases were seen on the CT scan, and cutaneous metastases were also present. The patient died 9 months later.

## Discussion

The spleen is tenth on the list of sites for solid tumor metastases [4-6,10]. Metachronous splenic metastasis from oesophageal cancer is a very unusual finding: thus far only four cases have been reported in the literature [9-14]. Despite the organ's high vascularity, the incidence of splenic metastasis in patients with solid tumors ranges from only 0.3% to 7.3%, and in over half of these cases the splenic involvement is part of disseminated disease involving three or more organs [4]. Primary cancers of the breast, lung, colon and rectum, ovary, and stomach are the ones most likely to metastasize to the spleen.

Isolated splenic metastases are very rare. Sileri et al. recently reported a case of a single splenic metastasis from colon cancer that appeared five years after surgery [8]. Several anatomic, hemodynamic, and immunologic hypotheses have been proposed to explain the rarity of splenic metastases, but none of them are fully convincing.

Consensus holds that metastases to the spleen are hematogenous and may be a part of generalized blood-borne dissemination. This assumption is largely based on the hypothesis by Marymount and Gross, who concluded that splenic metastases arise from circulating cancer cells that arrive via the organ's arterial blood supply [15]. Back in 1929, Wolgom suggested that tumor cells reaching the spleen might be destroyed by a humoral substance produced in the organ itself, the so-called splenic factor [7]. More recently, others have claimed that the spleen's resistance to metastases is related to periodic contractions of the capsule, which would keep the tumor cells in constant motion by forcing the blood from the sinusoids into the splenic veins [4-6]. Others have suggested that metastatic tumor cells arriving in the spleen would (like other foreign cells) undergo phagocytosis in the Billroth cords by the macrophages and tissue histiocytes [16].

The splenic lesions may be associated with vague non-specific symptoms or with pain or discomfort (like that reported by our patient). More frequently such condition are diagnosed incidentally on imaging studies performed during routine follow-up. The increasing availability and use of high-resolution modalities multi-slice CT and MRI has led to the identification of an increasing number of splenic metastases from different types of solid tumors, which previously escaped detection [17].

Our experience confirms that locoregionally controlled esophageal cancer can be associated with splenic metastases. Our patient's lesions were discovered during a routine follow-up visit after a 9-month disease free interval, and the patient had no clinical evidence of systemic disease at the time. The splenic metastases appeared as nonspecific lesions on abdominal CT and were promptly subjected to FNAC. Space-occupying lesions in this organ are more commonly related to other causes, but when the patient has a history of malignancy, the possibility that the splenic lesions are metastatic must be ruled out.

Splenic metastases are not a widely discussed problem. Because they are frequently associated with disseminated metastases, their presence is usually considered a sign of rapidly progressive disease that is no longer eligible for curative treatment. Noteworthy exceptions are cases occurring in patients with hematologic malignancies and those in which the splenic involvement is limited to a solitary lesion. The latter can sometimes be successfully managed with splenectomy, particularly when lesion onset occurs years after potentially curative surgical resection of the primary tumor. Further studies are necessary to define the prognostic impact of these lesions and their potential relation to the original histotype.

## Conclusion

Esophageal carcinoma is a rare cause of splenic metastases, and only four cases of isolated splenic metachronous secondaries have been reported thus far [3,9,10]. Given the unusual location of these lesions and our limited understanding of their behavior, we feel that splenectomy with en bloc lymph node dissection could help to stage the risk of further spread and to clarify if a wide systemic involvement is already present. Unfortunately a CT scan performed 3 months after surgery revealed multiple metastases to the liver and skin. The patient died 9 months later. Due to the small number of cases that have been reported, the role of adjuvant-combined modalities of treatment is unclear. Further exploration of these approaches might allow us to offer additional options for these unfortunate patients.

## Consent

Written informed consent was obtained from the next of kin of the patient for publication of study on January 2011. A copy of the written consent is available for review by the editor in Chief of this journal.

## List of abbreviations

FNAC: fine-needle aspiration cytology; COPD: chronic obstructive pulmonary disease; SSC: squamous cell carcinoma; CT: Computed tomography, MRI: magnetic resonance imaging; POD: post-operative day.

## Competing interests

The authors declare that they have no competing interests.

## Authors' contributions

IB: principle investigator who prepared, organized, wrote, and edited all aspects of the manuscript. JV: General surgeon who performed both operations and supported the work of principle investigator in preparing the manuscript. LC: supported the work of principle investigator in preparing the manuscript. GF: analyzed FNAC of the spleen and supported the work of principle investigator in writing and editing the manuscript. SB and VP: supported the work of principle investigator in writing and editing the manuscript. AV: chose imaging CT scan and supported the work of principle investigator in writing and editing the manuscript. MM and PD: They read, edited, and approved the final version of the manuscript. All authors read and approved the final version of the manuscript
